# An Account of the Successful Application of the Trepan in a Disease of the Tibia

**Published:** 1789

**Authors:** M. Verguin

**Affiliations:** Surgeon Major of the Naval Hospital and Inspector of the Royal College of Surgery at Toulon


V. *An Account of the ſucceſsful Application of the Trepan in a Diſeaſe of the Tibia. By M.* Verguin, *Surgeon Major of the Naval Hoſpital and Inſpector of the Royal College of Surgery at Toulon.* Vide *Journal de Medecine Militaire,* Tome VII. 8vo. Paris, 1788.

THE ſubject of this paper was a galley ſlave at Toulon, aged twenty-ſix years, who was brought to the naval hoſpital, in December, 1787, for the cure of a wound he had received, by the fall of a piece of timber, on his right leg.

The integuments of the anterior and middle part of the leg were much torn; and he complained of violent lancinating pain within the tibia, although no injury of the bone was perceptible externally.

It appeared, on farther inquiry, that about ſix years before this the patient had been wounded by a muſket ball in the calf of this leg; that three years after he had received a violent blow on the middle of the ſame leg, and that the wound occaſioned by this ſecond accident had not only been a long time before it healed, but had repeatedly broke out again, and that from from that period he had been ſubject to pain, more or leſs acute, in the interior part of the tibia.

When he came to the hoſpital the limb was conſiderably ſwelled, but the tenſion, under proper treatment, gradually ſubſided: the pain, however, within the tibia ſtill continued, and the wound poſſeſſed an extraordinary degree of ſenſibility.

Different topical applications, as well as internal remedies, were had recourſe to by our author during the ſpace of four months, but without ſucceſs. The wound was ill conditioned, and the painful affection of the tibia was not diminiſhed.

At this period, reflecting on all the circumſtances of the caſe, he determined to make an opening into the middle part of the tibia, in the manner recommended by ſome writers in caſes of ſpina ventoſa. He accordingly employed the trepan for this purpoſe. The piece of bone removed by the operation was very compact and of extraordinary thickneſs. Within the cavity of the bone at this part he found a ſmall quantity of a reddiſh fluid, ſurrounding three ſmall detached portions of bone which he extracted. One of theſe portions, he

 obſerves, obferves, was of the fize of a large lentile feed, and of an angular fhape; and the two others were irregularly fhaped, and fomewhat larger.

Within half an hour after the operation the pain with which the patient had been fo long afflicted ceafed. Suitable dreffings were applied to the wound; care was taken to regulate the health of the patient; and on the twenty-fourth day after the operation an exfoliation from the opening made in the bone by the trepan came away, and the cavity was foon after filled with a fubftance, which, in a fhort time, became as firm as the reft of the bone.

The operation was performed on the 30th of April; on the 13th of July the wound was healed; and the patient was difcharged from the hofpital the month following in perfect health.

